# Communication needs of cardiac surgery patients during their wake up process

**DOI:** 10.1186/2197-425X-3-S1-A923

**Published:** 2015-10-01

**Authors:** A Koyuncu, A Yava, FE Aslan, U Demirkılıç

**Affiliations:** Gülhane Military Medical Academy, Cardiovascular Surgery Unit, Ankara, Turkey; Hasan Kalyoncu University, School of Health Sciences, Department of Nursing, Gaziantep, Turkey; Acıbadem University, School of Health Sciences, Department of Nursing, İstanbul, Turkey

## Objectives

The aim of this study was to determine the mostly needed communication topics and define communicational needs of cardiac surgery patients during their wake up process.

## Methods

This was a descriptive and observational study implemented in a training and research hospital between 1 March and 1 August, 2014, among 132 volunteering patients. The descriptive data of patients were obtained from patient records and the patients themselves. The preferred communication method and topics of intubated patients were decided after the observations taken place during their wake up process. Beginning after two hours after their wake up, the patients who were capable to speak were questioned face to face interview about the sufficiency of the communication during the wake up process.

## Results

Seventy eight percent of volunteering patients had undergone coroner bypass surgery and sixty-eight percent were male. The communication method preferences of intubated patients during their wake up process were as follows; confirmation (48%), signing (35%), touching (09%), writing (05%), using communication cards (02%). The patients stated the needed topics during the process as; "I can't breathe, and take the tube out" (59%), "Did the operation finish?” Is it day or night? which day is today? What time is it?" (52%), "I have pain" (48%), "I am thirsty, I want water" (44%). Having been asked two hours after intubation about the sufficiency of communication during the process, patients stated that they experienced difficulties in building communication (41%), couldn't communicate because of weakness, tiredness and pain (%32), couldn't remember the process (21%), and didn't experience any problem in communication.

## Conclusions

The most frequent communication method patients used was approval, whereas the most frequent topics were about physiologic needs and information related to the surgery. Patients experienced difficulty in or no communication. As the result of this study it was considered that nurses should properly observe their patients, pay attention to their communication requests know the most frequent topics and improve their communicational skills.

## Grant Acknowledgment

We have no financial or other support for this study.
